# Molecular identification and phylogenetic analysis of GABA-producing lactic acid bacteria isolated from indigenous dadih of West Sumatera, Indonesia

**DOI:** 10.12688/f1000research.16224.1

**Published:** 2018-10-19

**Authors:** Lili Anggraini, Yetti Marlida, Wizna Wizna, Jamsari Jamsari, Mirzah Mirzah, Frederick Adzitey, Nurul Huda

**Affiliations:** 1 Graduate Program, Andalas University, Padang, West Sumatera, Indonesia; 2 Department of Nutrition and Feed Technology, Faculty of Animal Science, Andalas University, Padang, West Sumatera, Indonesia; 3 Department of Plant Breeding, Faculty of Agriculture, Andalas University, Padang, West Sumatera, Indonesia; 4 Department of Veterinary Science, University for Development Studies, Temale, Ghana; 5 School of Food Industry, Universiti Sultan Zainal Abidin, Kuala Nerus, Terengganu, 21300, Malaysia

**Keywords:** indigenous dadih, GABA, LAB, 16S rRNA gene, phylogenetic analysis

## Abstract

**Background:** Dadih (fermented buffalo milk) is a traditional Indonesian food originating from West Sumatra province. The fermentation process is carried out by lactic acid bacteria (LAB), which are naturally present in buffalo milk.  Lactic acid bacteria have been reported as one of potential producers of γ-aminobutyric acid (GABA). GABA acts as a neurotransmitter inhibitor of the central nervous system.

**Methods:** In this study, molecular identification and phylogenetic analysis of GABA producing LAB isolated from indigenous dadih of West Sumatera were determined. Identification of the GABA-producing LAB DS15 was based on conventional polymerase chain reaction. 16S rRNA gene sequence analysis was used to identify LAB DS15.

**Results:** PCR of the 16S rRNA gene sequence of LAB DS15 gave an approximately 1400 bp amplicon.  Phylogenetic analysis showed that LAB DS15 was *Pediococcus* *acidilactici*, with high similarity of 99% at 100% query coverage to *Pediococcus* *acidilactici *strain DSM 20284.

**Conclusions:** It can be concluded that GABA producing LAB isolated from indigenous dadih was *Pediococcus acidilactici*.

## Introduction

The non-proteinogenic amino acid γ-aminobutyric acid (GABA) is widely found in bacteria, animals, plants, and fungi ( Dhakal *et al*., 2012; Nonaka *et al*., 2017). GABA acts as a neurotransmitter inhibitor of the central nervous system ( Olsen & Li, 2012). It is formed by decarboxylation of L-glutamate, a reaction catalyzed by an enzyme that depends on the peridoxal phosphate of decarboxylated L-glutamate ( Murray *et al.*, 2003). Lactic acid bacteria (LAB) have been reported as a potential producer of GABA ( Seo *et al.*, 2013; Wu & Shah, 2017). LAB are generally regarded as safe and non-pathogenic microbes, and has been referred to as ‘generally recognized as safe’. Therefore, GABA-producing LAB can be used directly in functional foods ( Zhao *et al*., 2017). Some LAB can be found in the dairy industry for the production of cheese, yogurt, and other fermented milk products ( Yamada *et al.*, 2018).

Dadih (fermented buffalo milk) is an Indonesian traditional food originating from West Sumatra Province; it is an extremely popular dairy product in Bukittinggi, Padangpanjang, Solok, Lima Puluh Kota, and Tanah Datar, Indonesia ( Surono, 2015). Dadih is made from buffalo milk which is fermented in bamboo for 24–48 hours. The fermentation process is carried out by LAB which are naturally present in buffalo milk ( Rizqiati *et al*., 2015) and the environment ( Wirawati *et al*., 2017). Studies have found that, the LAB strains present in dadih are generally *Lactobacillus*, *Streptococcus*, *Leuconostoc* and *Lactococcus* ( Collado *et al*., 2007; Surono, 2003).

Extraction of DNA is a basic principle in molecular analysis and it is one of the success factors in DNA amplification that is used in the analysis of genetic characters ( Mustafa *et al.*, 2016). Polymerase chain reaction (PCR) and phylogenetic analysis based on 16S rRNA gene sequences have been used for successful identification of isolates from various fermented food products ( Malik *et al*., 2015). These molecular approaches have allowed *Lactobacillus* species to be reliably identified ( Henry *et al*., 2015). This research was conducted to identify and to characterize GABA producing LAB isolated from indigenous dadih of West Sumatera based on 16 S rRNA gene sequence analysis.

## Methods

### Sample

This study used lactic acid bacteria (LAB) DS15, a GABA-producing LAB isolated from dadih of West Sumatera origin. This bacterium was isolated previously according to the method described by Ali *et al*. (2009). The experiment was carried out at the Feed Technology Industry Laboratory, Faculty of Animal Science, Andalas University, West Sumatra, Indonesia. LAB DS15 was grown anaerobically in MRS medium (Merck, Darmstadt, Germany) at 30°C and stored for further analysis.

### Isolation of bacterial genomic DNA

Isolation of the total genome of LAB DS15 was done using Genomic DNA Mini Kit purchased from Invitrogen (PureLinkTM, USA) by following the manufacturer’s instructions. We used Lysozyme (PureLinkTM, USA) at a concentration of 20 mg/ml to break down the bacterial cell wall to improve protein or nucleic acid extraction efficiency.

### 16S rRNA gene amplification

Genomic DNA of LAB DS15 was used for amplification of 16S rRNA gene. Amplification was done using forward primer 63F (5'-CAG GCC TAA CAC ATG CAA GTC-3') and reverse primer 138R (5'-GGG CGG GGT GTA CAA GGC-3'). The reaction was carried out in a volume of 50 μl. The PCR mixture contained 22 μl of MQ, 25 μl DreamTaq Green DNA Polymerase (Thermo Fisher Scientific, USA), 1 μl of each forward and reverse primer (10 μM each, IDT synthesized) and 1 μl template. Amplification conditions were 5 minutes of preheating at 95°C, 30 seconds denaturation at 95°C, 30 seconds of primer annealing at 58°C, 1 minute extension step at 72°C and post cycling extension of 5 minutes at 72°C for 35 cycles. The reactions were carried out in a thermal cycler (Biometra's T-Personal Thermal Cycler, USA).

### Electrophoresis

PCR products were stored at 4°C for further examination using 1% agarose electrophoresis in 1X, 100 V TAE for 30 minutes **.** The DNA bands formed from electrophoresis process was visualized using UV transluminator.

### Sequence alignment of the 16S rRNA gene

Sequencing of the 16S rRNA gene was performed at the Laboratory of Medical Molecular Biology and Diagnostic, Indonesian Institute of Sciences, Jakarta. Sequencing results were edited (contig and peak chromatogram verification) using the SeqMan ^TM^ II program. Analysis of 16S rRNA sequences of LAB DS15 was performed using NCBI BLAST. Multiple alignment was done using the ClustalX 2.1 program. BioEdit version 7.2.5 in edit mode to see the absence of an inverted sequence and align the sequence length. Kinship visualization was done using the combined phylogenetic tree of the MEGA 7.0.20 program with the Neighbor-Joining hood method ( Saitou & Nei, 1987).

## Results and discussion

**Figure 1.**
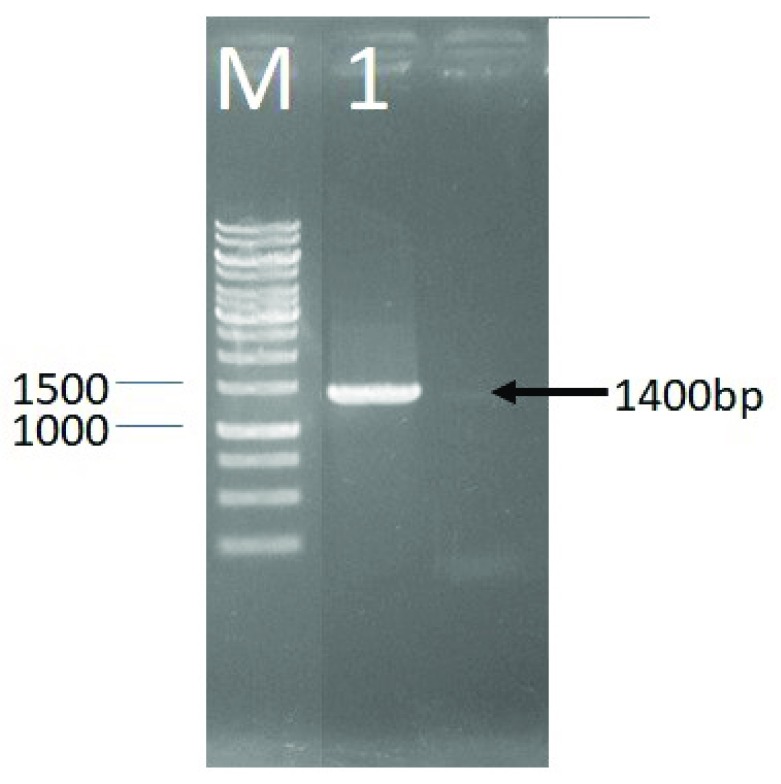
Agarose gel (1%) electrophoresis showing amplified 16S rRNA gene of LAB DS18. M, DNA marker; 1, PCR product of LAB DS18.

The identification of LAB DS15 to determine the strain was done based on 16S rRNA gene. The first step was amplification using PCR method, with the electrophoresis image shown in Supplementary File 1. The amplification process was carried out to obtain more copies of the 16S rRNA gene for the sequencing process. Analysis of sequencing results begun by aligning the base sequence of the 63F forward sequence and reverse 138R using the SegMan program. PCR of the 16S rRNA gene of LAB DS15 gave an approximately 1400 bp amplicon ( Figure 1).

Saitou & Nei (1987) indicated that the evolutionary history of organisms can be known using the neighbour-joining method. Organisms within the same taxa are normally clustered together in the phylogenetic tree and have better bootstrap values ( Felsenstein, 1985). In this study, we drew a phylogenetic tree to scale and determined the evolutionary distances using the p-distance method. A total of 26 nucleotide sequences and codon positions 1st + 2nd + and 3rd + noncoding were considered, using MEGA 7.0 as reported by Kumar *et al*. (2016) for evolutionary analyses.

DNA sequencing results were analyzed using NCBI BLAST. According to Willey *et al.* (2009), 16S rRNA sequencing looks at the similarity of isolates to those already available in GenBank; this is one molecular detection method that is ideal enough to know the kinship relationship between bacteria because the 16S rRNA sequence is a gene found in all microbes and is indispensable in maintain life. The 16S rRNA gene sequencing identified the LAB DS15 to belong to the genus *Pediococcus*, forming a well-defined cluster with *Pediococcus acidilactici.* This cluster was recovered in 100% of bootstrap analysis. *Pediococcus spp.* are widely described as probiotics ( Porto *et al*., 2017). Abbasiliasi *et al.* (2012) also found *Pediococcus acidilactici* in fermented milk products. *Pediococcus acidilactici* are important LAB which have been used as starter cultures in meat, vegetable and dairy fermentation causing characteristic flavor changes, improving hygiene and extending the shelf life of these products ( Mora *et al*., 1997; Porto *et al*., 2017).

**Figure 2.**
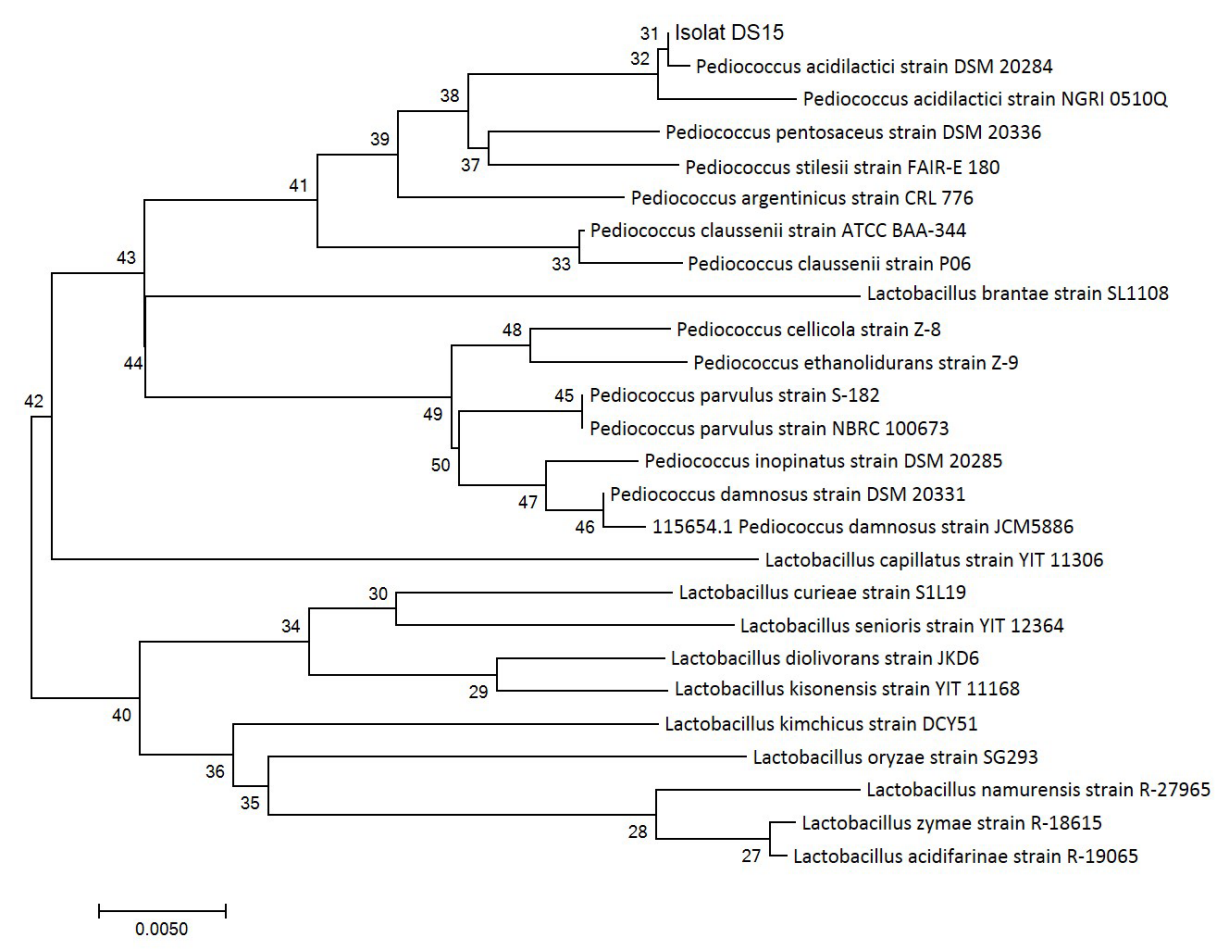
Phylogenetic tree of 16S rRNA gene of LAB DS18 using the neighbor-joining method.

**Figure 3.**
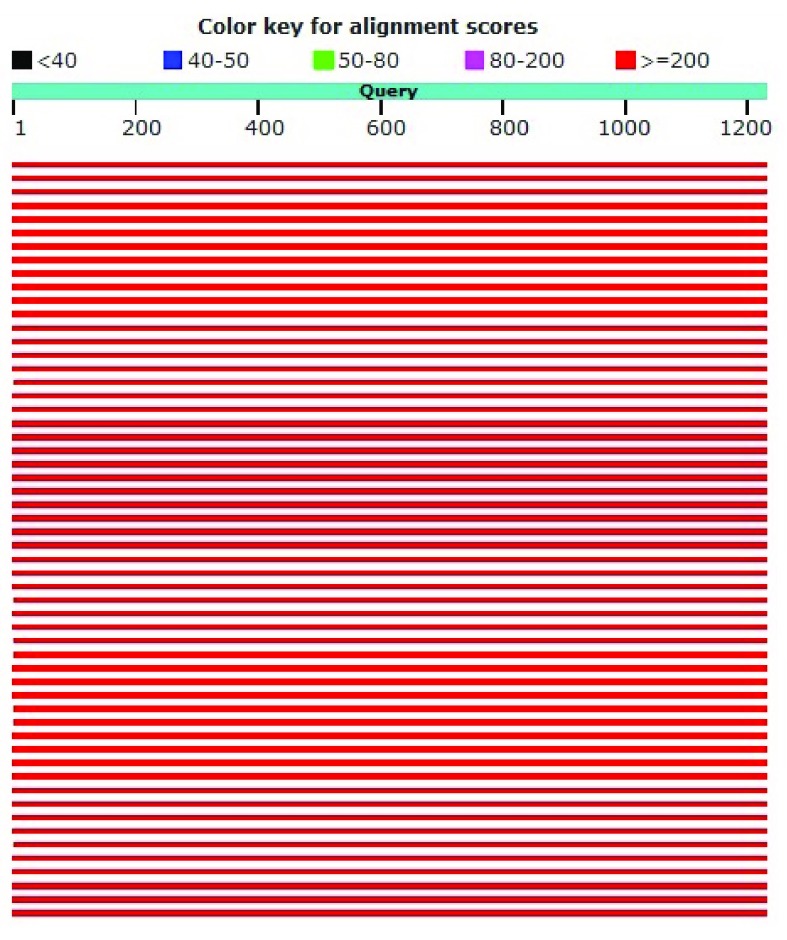
Graphic summary of DS18 and *Pediococcus acidilactici* strain DSM 20284.

A phylogenetic tree ( Figure 2) was constructed to determine the kinship relationship of LAB DS15. The phylogenetic tree is known to show a high consistency of the relationships between organisms. In this study, the isolate showed similarity of 99% at 100% query coverage to *Pediococcus acidilactici* strain DSM 20284. A value of 99% indicates that the isolate can be considered as the same species with *Pediococcus acidilactici* strain DSM 20284. The sequence of homology levels was high, as shown by the red color with a score of ≥200 ( Figure 3). From the results of this homology it can be concluded that the two sequences are the same and have an evolutionary relationship.

The next closest species for which a sequence alignment of at least 100% query coverage was observed were *Pediococcus pentosaceus* strain DSM 20336, *Pediococcus acidilactici* strain NGRI 0510Q and *Pediococcus argentini* strain CRL 776 at 98% similarity to the DS15 isolate. *Pediococcus stilesi* strain FAIR-E 180 showed 98% similarity with 99% query coverage. An alignment query result of 100% indicates a significant alignment, which means the search sequence in this study was identical with the identified genus, even at the species level.

## Conclusion

The PCR of 16S rRNA gene sequence gave an approximately 1400 bp amplicon for LAB DS15, isolated from indigenous dadih of West Sumatera. Phylogenetic analysis showed that LAB DS15 was *Pediococcus acidilactici,* with 99% similarity to *Pediococcus acidilactici* strain DSM 20284.

## Data availability

*Pediococcus acidilactici* strain DS32 16S ribosomal RNA gene, partial sequence, obtained during this study. GenBank accession number MH938236: http://identifiers.org/ncbigi/GI:1481059229.

## Supplementary material

**Supplementary File 1. Electrophoresis image of the PCR amplification product.**
