# Supplementary material for: Molecular identification and phylogenetic analysis of GABA-producing lactic acid bacteria isolated from indigenous dadih of West Sumatera, Indonesia
Source: F1000Res. 2019 Oct 17;7:1663. Originally published 2018 Oct 19. [Version 3] doi: 10.12688/f1000research.16224.3 (PMC7076337; doi:10.12688/f1000research.16224.3)
Supplement: Supplementary file 1 [file f1000research-7-22366-s0000.tgz › d83588a9-6818-45f6-9fe2-e7757e04bd9f_LAB_DS15_sequences_and_amplification_picture.doc]

Supplementary File 1.

Amplification picture

Amplification result of LAB DS15 is number 2

1kb DNA ladder	1	2	NTC

1400bp

1000

1500
