# Supplementary material for: Molecular identification and phylogenetic analysis of GABA-producing lactic acid bacteria isolated from indigenous dadih of West Sumatera, Indonesia
Source: F1000Res. 2019 Oct 17;7:1663. Originally published 2018 Oct 19. [Version 3] doi: 10.12688/f1000research.16224.3 (PMC7076337; doi:10.12688/f1000research.16224.3)
Supplement: Peer review history

# Peer review: Reviewer response for version 3

**DOI:** 10.5256/f1000research.22366.r56703

**Published:** 2020-03-12

**Authors:** Usman Pato

**Affiliations:** 1 Faculty of Agriculture, Riau University, Pekanbaru, Indonesia

**INTRODUCTION**

1. In general, the introduction is relatively good but needs to be added by the results of research from Hosono et al 1989 ^1^ and Wirawati et al., 2019 ^2^ about micflora in dadih
2. In the introduction, the author needs to explain in more detail the role of GABA produced by LAB and other organisms 

 
**METHODS **

1. An explanation should be added as to why only to choose the DS15 strain producing GABA in this study.
2. It is necessary to add the reference methods used in the 16S rRNA gene amplification analysis and electrophoresis process

 
**RESULTS AND DISCUSSION**

 The results are well presented and discussed systematically because the authors used only one strain.

   

**REFERENCES**

 The author needs to add references as a follow-up to suggestions for improvement in the introduction and method of this paper

**The strength of this paper**

 The strength of study is the first research to report on GABA-producing LAB from dadih and local fermented milk products from Indonesia

**The weakness of this paper**

 One of the weaknesses of this study is that the authors only used one LAB dadih isolate (strain DS15) so that no comparative data were produced and the discussion was relatively limited.

I confirm that I have read this submission and believe that I have an appropriate level of expertise to confirm that it is of an acceptable scientific standard.

## References

[1] : Microbial flora in dadih a traditional fermented milk in indonesia. *Life, Earth*.

[2] : Diversity of lactic acid bacteria in dadih produced by either back-slopping or spontaneous fermentation from two different regions of West Sumatra, Indonesia. *Vet World*.2019;12(6) : 10.14202/vetworld.2019.823-829 823-829 10.14202/vetworld.2019.823-829 31440000PMC6661478

# Peer review: Reviewer response for version 3

**DOI:** 10.5256/f1000research.22366.r55312

**Published:** 2019-11-25

**Authors:** Qinglong Wu

**Affiliations:** 1 Texas Children's Microbiome Center, Baylor College of Medicine, Houston, TX, USA

I did not see any improvements of scientific value that have been made in the revision.

I confirm that I have read this submission and believe that I have an appropriate level of expertise to state that I do not consider it to be of an acceptable scientific standard, for reasons outlined above.

# Peer review: Reviewer response for version 2

**DOI:** 10.5256/f1000research.19627.r48482

**Published:** 2019-06-11

**Authors:** Sahilah Abd Mutalib

**Affiliations:** 1 Centre for Biotechnology and Functional Food, Faculty of Science and Technology, Universiti Kebangsaan Malaysia (UKM), Selangor, Malaysia

1. Introduction - fairly good and can be improved

 i. Dadih from **Indonesia has- *Lactobacillus*, *Streptococcus*, *Leuconostoc* and *Lactococcus - *How did they identify the bacteria? Biochemical tests or using molecular approaches?

 ii. Is there any data on dadih from Malaysia as well for comparison.

 2. Methods - can be improved

 2.1 Sample - Subtopic sample suggested to change - Bacterial strain

 The month and year of the bacterium should be mentioned for ex: in June 2009. Why too long to continue the partial sequence 16s rRNA analysis?

 2. 2 Isolation of bacterial genomic DNA

 We used lysozyme-change to "Twenty(20) mg/ml of lysozyme was used to break down ...."

 Please state where did you keep the genomic DNA. Example in -20 ^o^C freezer or 4 ^o^C refrigerator prior analysis

 2.3 16S rRNA gene amplification

 Please state the **reference** after forward and reverse primers is mentioned.

 2.4 Electrophoresis

 1% change to 1% (w/v)

 in 1x change to1x TAE, 100 V

 The marker should be mentioned in this section, 1 Kb ladder? What kind of dye did you used? Red dye, syber green, ethidium bromide? State their brand as well

 Results and discussion

 Good - due to a single strain/isolate was studied, thus, the explanation is straight forward.

I confirm that I have read this submission and believe that I have an appropriate level of expertise to confirm that it is of an acceptable scientific standard.

# Response

**Published:** 2019-08-14

**Authors:** Nurul Huda

**Affiliations:** Universiti Malaysia Sabah, Malaysia

1. Introduction - fairly good and can be improved

 i. Dadih from Indonesia has- *Lactobacillus*, *Streptococcus*, *Leuconostoc *and *Lactococcus - *How did they identify the bacteria? Biochemical tests or using molecular approaches?

 They identify bacteria with a molecular approach using the 16SsRNA technique

 ii. Is there any data on dadih from Malaysia as well for comparison.

 No, we don’t have data on dadih from Malaysia.

 2. Methods - can be improved

 2.1 Sample - Subtopic sample suggested to change - Bacterial strain

 The month and year of the bacterium should be mentioned for ex: in June 2009. Why too long to continue the partial sequence 16s rRNA analysis?

 Bacterial strains isolated in July 2017.

 We did this isolation based on the method of Ali *et al., *(2009), not isolates from the author.

 2. 2 Isolation of bacterial genomic DNA

 We used lysozyme-change to "Twenty (20) mg/ml of lysozyme was used to break down ...."

 Please state where did you keep the genomic DNA. Example in -20 C freezer or 4 C refrigerator prior Analysis

 We keep the genomic DNA in 4 ^o^C refrigerator.

 2.3 16S rRNA gene amplification

 Please state the **reference **after forward and reverse primers is mentioned.

 We got reference for forward primer 63F (5'-CAG GCC TAA CAC ATG CAA GTC-3') and reverse primer 1387R (5'-GGG CGG GGT GTA CAA GGC-3') from Laboratory of Medical Molecular Biology and Diagnostic, Indonesian Institute of Sciences, Jakarta, Indonesia.

 2.4 Electrophoresis

 1% change to 1% (w/v)

 Ok we will change  

 in 1x change to1x TAE, 100 V

 Ok we will change 

 The marker should be mentioned in this section, 1 Kb ladder? What kind of dye did you used? Red dye, syber green, ethidium bromide? State their brand as well

 We used 1 Kb Plus DNA Ladder (ThermoFisher Scientific)

 Results and discussion

 Good - due to a single strain/isolate was studied, thus, the explanation is straight forward.

 Thank You.

# Peer review: Reviewer response for version 2

**DOI:** 10.5256/f1000research.19627.r46293

**Published:** 2019-04-08

**Authors:** Jagadish Mahanta

**Affiliations:** 1 Regional Medical Research Centre, Indian Council of Medical Research (ICMR), Dibrugarh, Assam, India

Authors wanted to identify and characterize GABA producing LAB isolated from “Dadih”.

1. However, authors have taken a strain already isolated and identified in 2009. Authors have not mentioned anything about the gap in the previous research that necessitated undertaking the present exercise. Authors may clarify the issue.
2. Authors have done elaborate molecular testing and phylogenetic analysis of the bacteria taken from the stock. Authors should elaborate the achievement of this exercise.
3. As emphasized by the authors, they should elaborate, about characterization and GABA production potential of the strain
4. Authors should elaborate on the novelty of the study.

I confirm that I have read this submission and believe that I have an appropriate level of expertise to confirm that it is of an acceptable scientific standard, however I have significant reservations, as outlined above.

# Response

**Published:** 2019-04-10

**Authors:** Nurul Huda

**Affiliations:** Universiti Malaysia Sabah, Malaysia

Authors wanted to identify and characterize GABA producing LAB isolated from “Dadih”.

 1. However, authors have taken a strain already isolated and identified in 2009. Authors have not mentioned anything about the gap in the previous research that necessitated undertaking the present exercise. Authors may clarify the issue.

 Exploration of isolates from dadih has been carried out, but no studies have used these isolates as GABA producers. In this study we obtained DS15 isolate from the isolation of various fermented foods which had the highest GABA production. We did this isolation based on the method of Ali *et al.,* (2019), not isolates from the author.

 2. Authors have done elaborate molecular testing and phylogenetic analysis of the bacteria taken from the stock. Authors should elaborate the achievement of this exercise.

 The result of BLAST at the NCBI GenBank site from the sequences showed that DS15 isolate were *Pediococcus acidilactici*. Based on the phylogenetic tree, DS15 has a 99% similarity or homology with *P. acidilactici* DSM 20284, with the difference of one base pair. The next closest species were *P. acidilactici* NGRI 0510Q, *P. pentosaceus* DSM 20336 and *P. argentinicus* CRL 776 with 98% similarity. *P. stilesi* FAIR-E 180 shows 98% similarity with 99% query coverage.

 3.  As emphasized by the authors, they should elaborate, about characterization and GABA production potential of the strain.

 We have carried out quantitative screening on some of the isolates we obtained from dadih, and we found that DS15 isolates produced the highest amount of GABA. Data and discussion are used in another publications.

 4. Authors should elaborate on the novelty of the study.

 The novelty of this study was the use of bacterial isolates from dadih as a GABA producer.

# Peer review: Reviewer response for version 2

**DOI:** 10.5256/f1000research.19627.r46425

**Published:** 2019-04-01

**Authors:** Qinglong Wu

**Affiliations:** 1 Texas Children's Microbiome Center, Baylor College of Medicine, Houston, TX, USA

The authors detailed the 16S rRNA gene-based to be a good lab protocol without demonstrating any scientific value. There is no experimental data to support the GABA production from isolate DS15. They have to present the GABA data in terms of GABA yield under defined fermentation conditions. Meanwhile, they have to demonstrate the pathway in isolate DS15 that is responsible for GABA biosynthesis and GABA export in this strain.

 Secondly, the authors just use one isolate to achieve the claim "GABA producing LAB isolated from indigenous dadih was *Pediococcus acidilactici"*. This is not a rigorous way.

 Here are my questions:

1. What is the level of GABA in dadih?
2. How is GABA production capacity of DS15?
3. There is no massive bacterial isolation from dadih; neither no microbial community profiling for dadih, nor pathway identification of GABA production for microbial community of dadih; so one isolate from dadih does not mean anything.

I confirm that I have read this submission and believe that I have an appropriate level of expertise to state that I do not consider it to be of an acceptable scientific standard, for reasons outlined above.

# Response

**Published:** 2019-04-10

**Authors:** Nurul Huda

**Affiliations:** Universiti Malaysia Sabah, Malaysia

1. What is the level of GABA in dadih?

We don't count the amount of GABA on dadih. We did not count the amount of GABA produced in dadih.  GABA is produced by lactic acid bacteria of dadih origin but not the dadih, so we don’t count or determine GABA level of dadih

1. How is GABA production capacity of DS15?

GABA production capacity of DS15 was 49.365 mg/L

1. There is no massive bacterial isolation from dadih; neither no microbial community profiling for dadih, nor pathway identification of GABA production for microbial community of dadih; so, one isolate from dadih does not mean anything.

In this study, we isolated bacteria from various fermented food products (dadih, ikan budu, asam durian and tape singkong), determined their GABA producing ability and selected the isolate with the highest GABA production for further identification. The results of isolation and characterization are explained in other articles. The distribution of LAB isolates from the indigenous West Sumatera fermented food (dadih only) is;

1.  Origin from Aiadingin area.  Number of isolate 131; Number of LAB isolate 125; Number of GABA producing LAB isolate 23.
2. Origin from Sijunjung area.  Number of isolate 166; Number of LAB isolate 93; Number of GABA producing LAB isolate 19.
3. Origin from Solok area.  Number of isolate 100; Number of LAB isolate 96; Number of GABA producing LAB isolate 19.

In total, from 3 areas, number of isolate 397; number of LAB isolate 314; number of GABA producing LAB isolate 62.
